# Uncovering the Concerns and Needs of Individuals with Celiac Disease: A Cross-Sectional Study

**DOI:** 10.3390/nu15173681

**Published:** 2023-08-22

**Authors:** Maialen Vázquez-Polo, Virginia Navarro, Idoia Larretxi, Gesala Perez-Junkera, Arrate Lasa, Silvia Matias, Edurne Simon, Itziar Churruca

**Affiliations:** 1 Gluten 3S Research Group, Department of Nutrition and Food Science, Faculty of Pharmacy, University of the Basque Country UPV/EHU, 01006 Vitoria-Gasteiz, Spain; maialen.vazquez@ehu.eus (M.V.-P.); idoia.larrechi@ehu.eus (I.L.); gesala.perez@ehu.eus (G.P.-J.); arrate.lasa@ehu.eus (A.L.); silvia.matias@ehu.eus (S.M.); edurne.simon@ehu.eus (E.S.); itziar.txurruka@ehu.eus (I.C.); 2 Bioaraba, Nutrition and Food Safety Group, 01009 Vitoria-Gasteiz, Spain; 3 Centro Integral de Atención a Mayores San Prudencio, 01006 Vitoria-Gasteiz, Spain

**Keywords:** celiac disease, gluten-free diet, health education, nutritional education

## Abstract

The treatment for celiac disease (CD) involves a strict gluten-free diet, which can be challenging and lead to questions for patients. Pinpointing these uncertainties can enable the creation of efficient educational resources. In this study, a questionnaire was specifically designed to gain insights into the knowledge, concerns, and needs of individuals with CD and their supporters. The questionnaire was distributed through the Instagram social network and received adequate responses from 300 participants, 258 (86%) being female and 152 (50.7%) falling in the age range of 25–44 years. The concerns of individuals with celiac disease and celiac supporters were rated on a 1–4 scale, with a mean score of 3.5 indicating significant concern. A total of 255 (85%) of all participants expressed that their principal concern was the social limitations they faced, such as difficulties in eating out and sharing food with others. Every participant evaluated their overall disease knowledge, averaging at 2.92 out of 4, indicating a reasonable level of awareness. When asked if they believed that improving general knowledge about CD in the general population would enhance their quality of life, the vast majority responded affirmatively. This finding underscores the importance of not only educating individuals with CD but also reaching out to the wider population, especially those who have a direct impact on the daily lives of individuals with CD, such as family members, friends, and food service providers.

## 1. Introduction

Celiac disease (CD) is an immune-mediated disorder that occurs in genetically susceptible individuals who consume gluten and related prolamins. It is characterized by the gradual deterioration of the intestinal villi following the ingestion of gluten [1]. The prevalence of CD has significantly increased in the last few decades at a rate of 7.5% annually [2,3], but its exact prevalence is still uncertain. It is often undiagnosed, with a ratio of from 1:3 to 1:5 between diagnosed and undiagnosed cases [4]. However, in most Western countries, it affects approximately 1–2% of the population [5,6], although there are variations among different countries and regions [2,5,7,8]. It is more common in women than in men and in children than in adults [6].

The gastrointestinal symptoms of CD can manifest as follows [9]:

- Diarrhea,
- Steatorrhea,
- Abdominal pain,
- Vomiting.

Moreover, there are some potential extraintestinal manifestations [9]:

- Anemia,
- Osteopenia,
- Osteoporosis,
- Reproductive disorders,
- Short stature,
- Asthenia,
- Dental enamel hypoplasia.

Currently, the only recognized and effective treatment for the disease is a strict and lifelong gluten-free diet (GFD), which involves excluding wheat, rye, barley, and related hybrids such as kamut and triticale from the diet [10]. Determining whether food contains gluten can be challenging due to cross-contact. Products that are naturally gluten-free, like fruits and vegetables, may come in contact with gluten before they are consumed or during their preparation and serving processes [11].

The practical challenges of managing a GFD are further complicated. Long-term adherence to a GFD can lead to various challenges such as anxiety in school or work settings, difficulties in social situations, ongoing clinical symptoms, and changes in body composition. Consequently, a significant consequence of this treatment is a decline in quality of life [12].

Several studies have noted that individuals with CD often experience feelings of being different and excluded, as it can be challenging to eat out, ensure gluten-free food options, and avoid cross-contact with gluten-containing foods [13,14,15,16,17]. Moreover, it has been described that people with CD may suffer from psychiatric disorders, such as anxiety or depression [18,19,20]. Certain factors may be attributed to the disease itself and its biochemical effects, while others are related to a patient’s subjective perception of the disorder and the GFD used to manage it. Difficulties in purchasing and preparing food, along with related costs within a family unit, can potentially contribute to feelings of anxiety according to Zingone et al. [19]. While some psychological problems may improve over time after diagnosis, as patients gain knowledge about the condition, it seems that many individuals continue to have concerns about managing a GFD and struggle to adhere to it, particularly in social situations and when going out [19].

In fact, a study revealed that social phobia, as measured by the Liebowitz Social Anxiety Scale, was significantly higher in individuals with CD [21]. Hallert et al. also assessed perceived worries, restrictions, and subjective outcomes in individuals with CD [22]. At 10 years after diagnosis, women expressed more concerns about how the disease affected their ability to socialize with friends and their need to abstain from important aspects of life.

Actually, the general population’s knowledge of CD is low, and this situation is responsible for part of the aforementioned problem. Kameswari et al. observed this in a study with dental students. It was found that only approximately 22.77% of the postgraduates were aware of CD [23]. In another study conducted by Dembinski et al. among medical students and medical professionals, they found that nearly half (46%) of the participants surveyed were unaware of the potential risk of nutritional deficiencies in individuals with CD [24]. The understanding of CD among individuals with a background in health sciences is often limited, so it is likely that the general population without a health science background has even less knowledge about the condition. This is reflected in other studies, such as one published in 2021 and carried out in Turkey. It concluded that the level of public awareness of the situation of people with CD was low [25].

In contrast, the level of knowledge about CD among catering industry staff has shown mixed results. While overall awareness of the disease is generally high and has shown improvement in recent years, there are still areas where gaps exist, such as accurately reporting the availability of gluten-free food options on menus or other forms of documentation [26,27].

In addition to the general population, individuals with CD might also find themselves with limited insight to their condition due to inadequate counseling and follow-up from healthcare professionals [27,28]. For example, in a study carried out by Leffler et al., a significant number of individuals expressed dissatisfaction with the services offered by their healthcare team in assisting them with the management of CD [29]. Another study by Riznik et al. revealed that patients’ knowledge about CD was unsatisfactory, with nearly one-fifth of patients scoring poorly on a section of a questionnaire related to disease treatment. Interestingly, parents exhibited higher knowledge levels compared to adults and children with CD, particularly regarding treatment and follow-up, potentially due to their responsibility for their child’s meals and heightened vigilance [30]. However, other studies have observed poor parental knowledge and attitudes toward CD management [31,32]. In a recent study by Sahin et al., similar findings were reported, highlighting low levels of knowledge and awareness among patients and their caregivers concerning CD [33].

For all these reasons, it is important to make progress in nutritional education, not only for patients but also for society in order to advance in the understanding and management of the disease as a whole. To this end, detecting the real concerns, needs, doubts, and difficulties of the population with CD is the starting point, and this is precisely the aim of this study.

## 2. Materials and Methods

### 2.1. Study Design and Instruments

A specific questionnaire was designed with the aim of detecting the knowledge, concerns, and needs of the celiac community. It was designed to be answered by people with CD or individuals caring for people with CD (e.g., parents or guardians of people with CD). The survey included either 16 questions for participants with CD or 18 questions for supporters (with two additional questions to define their relationship with the former). It was designed for online completion and featured multiple answer choices in order to achieve the highest possible accuracy in the answers (Appendix A). The following questions were asked about CD: date of diagnosis of participant, presence of symptoms (which and how often), general concerns about the disease, and adherence to a GFD. Participants were also asked the following about their knowledge of CD and GFDs: how they perceived their knowledge of the subject, what topic they would like to know more about, and where they usually look for information. Finally, they were asked about how they behaved when eating out.

The questionnaire was distributed through our GLUTEN3S research team’s Instagram account (https://www.instagram.com/gluten3s/ (accessed on 22 August 2023), and sampling was carried out using the snowball method. This method has been variously defined before [34] and consists of one respondent passing the questionnaire to another individual and the latter to another, thus growing the sample into a rolling snowball. In order to ensure greater dissemination, it was also shared through the Instagram account of a Spanish journalist specializing in health who actively works on CD (Celicidad) [35] and whom many experts on the subject collaborate with, such as doctors, dieticians/nutritionists, psychologists, etc. The collaboration was altruistic. Instagram was chosen because it is one of the most used platforms by people with CD [36] and because the research team had already used this social network for scientific dissemination purposes. However, it is crucial to have a substantial platform for disseminating information in order to reach potential participants effectively. Thus, it was concluded that an individual with expertise in social networks and, specifically, in the field of health, particularly CD, would be the most suitable person to aid in the dissemination process.

Prior to the start of the study, all participants agreed to participate in the study by signing an informed consent form. Prior to filling out the questionnaire, participants were required to review a document that outlined the purpose and objectives of the study, as well as their responsibilities, and to provide explicit authorization and consent to participate. This included granting permission for their data to be used in the project. Additionally, contact information for the researchers was provided to ensure accessibility for any further inquiries or concerns. The study was approved by the Ethics Committee for Human Research of the University of the Basque Country, UPV/EHU (M10/2020/081).

### 2.2. Statistical Analysis

IBM SPSS Statistics for Windows, version 28.0 (IBM Corp., Armonk, NY, USA), was used for the statistical analysis of the data. Participants who did not complete the questionnaire were excluded from the analysis. This decision was made because these participants only answered the initial questions related to sample characteristics and did not provide any additional information required for the study.

The descriptive analysis was carried out by means of a study of frequencies and percentages. Responses were divided into two groups: those from CD sufferers (celiac group) and those from non-sufferers (relatives, friends, or caregivers; supporter group). Both groups were presented with identical sets of questions (only two more questions for the group of supporters in order to establish their relationship with the other group), and the analysis of the results followed the same methodology.

Chi-squared tests were used to compare the qualitative responses of both groups. The Mann–Whitney U-test was used to compare both groups when the responses were quantitative (Likert 1–4 scales; see Table A1 in the Appendix A). Results were considered significant when *p* < 0.05 (confidence interval of 95%).

## 3. Results

### 3.1. Sample Characteristics

The questionnaire was responded to by 305 people. Five of those respondents did not complete the whole questionnaire and, therefore, they were withdrawn from the analysis. From the 300 participants who adequately completed the questionnaire, 86% were female, 13.7% were male, and 0.3% identified themselves with another gender option. A total of 10% of participants were aged 18–24 years, 50.7% were 25–44 years, 37.3% were 45–64 years, and 2% were over 65 years.

A total of 89% of respondents resided in Europe, 9% in South America, 1% in North America, and 1% in Central America.

In terms of educational level, 7.3% had a primary education (preschool education, special education, and similar studies), 12.3% had a secondary education (baccalaureate, secondary school, official language studies, university entrance, and similar), 19.3% had a professional education (professional training modules, first- and second-degree vocational training, intermediate and advanced vocational training cycles, other professional or artistic studies), 16.3% had a medium–high education level (technical engineering studies, industrial expertise, first-cycle university studies, specialization studies in secondary education, and others at the same level), and 44.7% had a higher education level (bachelor’s degree, higher engineering, postgraduate, master’s degree, doctorate, and specialization).

Among the participants, 74.3% identified themselves as having celiac disease (self-reported, celiac group), while 25.7% identified as not having celiac disease (supporter group). Among the latter, with regard to their relationship with the former, 84.4% were cohabitants, 11.7% were non-celiac family members, and 3.9% were friends. Regarding their role in the care of a person with CD, 72.7% of the respondents were caretakers, 14.3% were occasional caretakers, and 13.0% were not caretakers.

Among the persons with CD, 22.9% were diagnosed less than one year ago, 43.5% were diagnosed 1–5 years ago, 18.8% were diagnosed 5–10 years ago, and 14.8% were diagnosed more than 10 years ago. Among supporters, 24.7% reported that their close person with CD was diagnosed less than one year ago, 54.5% were diagnosed 1–5 years ago, 15.6% were diagnosed 5–10 years ago, 1.3% were diagnosed more than 10 years ago, and 3.9% did not know.

### 3.2. Dietary Adherence Reported by Respondents

Adherence to diet was measured through the question “Do you follow a strict gluten-free diet?” and had the following response options: “Yes, always, no exceptions”; “Yes, although I sometimes consume it unintentionally”; “Yes, although I sometimes consume it intentionally”; “No, I do not follow a strict gluten-free diet”; and “I do not know”. Only one answer option could be chosen. Supporters were asked to respond in reference to their close person with CD. Results are shown in Table 1.

### 3.3. Symptomatology Reported by Respondents

Participants’ symptomatology was studied through two questions: “Do you have symptoms related to celiac disease?” and “If you have symptoms, how recurrent are they?” On the one hand, the first question had four answer options: “Yes, I have gastrointestinal symptoms (diarrhea, gas, bloating, etc.)”; “Yes, I have extraintestinal symptoms (anemia, dermatitis herpetiformis, etc.)”; “Yes, I have gastrointestinal and extraintestinal symptoms”; and “No, I have no symptoms”. Only one option could be chosen. Results are shown in Table 2.

There were significant differences between the symptoms reported by the celiac and the supporter groups (chi-squared test: *p* < 0.001). The latter group most frequently chose the answer “the person with CD close to me has no symptoms”.

On the other hand, the second question had six answer options: “Never”; “Very occasionally”; “A few times a year”; “Every month”; “Every week”; and “Every day”. Only one answer option could be chosen. Supporters responded in reference to their close person with CD. Results are shown in Table 3.

There were significant differences between the frequency of the symptoms reported by the celiac and supporter groups (chi-squared test: *p* < 0.001). The supporter group most frequently chose the answer “the person with CD close to me never or very occasionally has symptoms”.

### 3.4. Concerns about Celiac Disease 

Regarding the respondents’ concern about celiac disease, they were asked the following question: “From 1 to 4, how would you define your level of concern about the disease?” On a scale from 1 (not at all) to 4 (very much), they defined their concern with a mean of 3.5 (standard deviation of 0.738). Specifically, the celiac group had a mean of 3.42 (standard deviation of 0.795), and the supporter group had a mean of 3.73 (standard deviation of 0.477). There were statistically significant differences between the two groups (*p* < 0.05 in Mann–Whitney U-test). The caregiver group expressed more concern than the celiac group.

To find out what specifically concerned them most, they were questioned as follows: “What worries you most on a day-to-day basis?” The six response options were “Social limitations: eating out, attending events or celebrations, sharing food with people, etc.”; “Feeling socially excluded”; “Making the gluten-free diet healthy and balanced”; “Cross-contact”; “Following a 100% gluten-free diet”; and “Other”. More than one answer option could be chosen. Results are shown in Table 4.

Among the people who chose “Other” and openly developed their answer, the most repeated concerns were the following: misinformation by the general population, as well as by relatives, doctors, and catering professionals; the high price of gluten-free products; and the nutritional value and safety of the ingredients used to create gluten-free products.

There were significant differences between the main concerns of the celiac and supporter groups (chi-squared test: *p* < 0.001). For both groups, the major concern was social limitations followed by cross-contact, but the response rate was significantly higher in the group of people with CD.

### 3.5. Uncomfortable Aspects of Social Gatherings for Individuals with CD

To find out what made people with CD most uncomfortable at social gatherings, they were asked the question directly: “What makes you most uncomfortable at a social gathering?” There were six response options: “Having to give explanations of CD”; “Feeling misunderstood because people underestimate the situation”; “Having to make sure no one contaminates my food”; “Having to bring my own food”; and “Other”. Only one answer option could be chosen. Results are shown in Table 5.

Among the people who chose the option “Other”, nine answered “all or several answers above”. Four people developed their concern; in two of the cases, their answers were similar to “Having to make sure that no one contaminates my food”. Two cases selected “Having to give explanations of CD”. One person reported not feeling uncomfortable in these situations. No statistically significant differences were found between the two groups (*p* = 0.756 in chi-squared test).

### 3.6. Self-Perceived Knowledge about CD in Celiac People and Knowledge Updating

People who took part in the survey were asked how much they felt they knew about CD (Question: “How much do you think you know about CD?”). Among people with CD, 1.8% rated their knowledge with 1 point, 22.4% with 2 points, 57.4% with 3 points, and 18.4% with 4 points. On the other hand, in the caregiver group, 5.2% rated their knowledge with 1 point, 20.8% with 2 points, 59.7% with 3 points, and 14.3% with 4 points. No statistically significant differences were found between the two groups (chi-squared test: *p* = 0.367). In the overall respondent group, 2.7% rated their knowledge as 1 point, 22.0% as 2 points, 58.0% as 3 points, and 17.3% as 4 points.

Although the level of self-perceived knowledge was quite high in both groups, most participants reported seeking information from a variety of sources on a regular basis. To the question “Are you usually looking for information about CD?” the possible answers were “Yes, through the Celiac Association, doctors, dieticians/nutritionists, and other professionals”; “Yes, through the social networks of professionals”; “Yes, through the social networks of people who talk about their experiences”; and “No, I think I already know enough”. More than one answer option could be chosen. Results are shown in Table 6.

### 3.7. Actions That People with CD Take When Eating out in Restaurants

In order to find out how people with celiac disease behave when eating out in a restaurant, they were asked the next question: “How often have you done these actions when going to a restaurant?” In addition, they were exposed to four situations: “Notify the restaurant about the disease to make sure that gluten-free food is available”; “Notify about the disease to avoid cross-contact”; “Check that the restaurant is certified gluten-free”; and “See first-hand if the restaurant has good hygiene and can carry out a gluten-free menu”. They were asked to mark how often they performed the following actions on a Likert scale: 1 (never), 2 (sometimes), 3 (many times), or 4 (always). Results are shown in Table 7.

### 3.8. Foods That Raise Suspicion among Individuals with CD

In an attempt to find out which foods people with CD were wary of, they were given a choice of products from the following list: “Gluten-free cereals”; “Generic foods that naturally do not contain gluten (dairy products, legumes, packaged vegetables, etc.) and are not labeled as gluten-free”; “Processed food with a long list of ingredients”; “Spices”; “Non-food: parapharmacy products, medicines, cosmetics, etc.”; and “Others”. They could choose from all the options they considered. Results are shown in Table 8.

Among the people who ticked the option “Other” (15 participants), five stated that the most suspicious foods were those that were mislabeled or did not bear official stamps. Four indicated teas or infusions. Four mentioned specific food names. One answer was “Non-food: parapharmacy products, medicines, cosmetics, etc.”, and a last answer was left blank.

No statistically significant differences were found between the celiac and supporter groups (*p* = 0.313 in chi-squared test).

### 3.9. The Information People with CD Seek

As well as understanding how people with CD perceived their own knowledge of the disease, it is valuable to explore the specific areas about which they would like to learn more. The survey presented eleven different topics to participants, who were asked to rate their level of interest on a Likert scale ranging from 1 to 4: “1—I am not interested at all”; “2—I am a little interested”; “3—I am quite interested”; and “4—I am very interested”. Results are shown in Table 9.

### 3.10. Perceptions of People with CD: Can the Improvement of Knowledge Have a Positive Impact on the Quality of Life?

Respondents were asked about their point of view on whether knowledge of CD in the general population would improve their own quality of life. They were presented with a number of response options and asked to select the most appropriate one. The answer options were as follows: “Yes, considerably”; “No”; and “Not sure”. Only one answer option could be chosen. Results are shown in Table 10.

## 4. Discussion

Limited research has been conducted on the knowledge, concerns, and needs of the celiac community, and this study marks one of the initial efforts in this area. Al Sarkhy conducted a study that examined the utilization of social networks among individuals registered in the Saudi Celiac Patients Support Group (SCPSG) [36]. The study revealed that 96% of patients relied on social media platforms to manage their disease, suggesting that social media could serve as a valuable source of representative information for the celiac community [36,37]. Similar findings have been observed in larger studies conducted on other digestive disorders [38,39]. Additionally, the study of Al Sarkhy gathered insights on usage patterns, limitations, and opinions of individuals with CD and their caregivers toward social media. To ensure a comprehensive representation of the population, it is important to involve individuals who are closely associated with persons with CD, particularly caregivers, as online questionnaires may not be suitable for children under 18 years of age [40]. It is worth mentioning that the responses from supporters and participants with CD were comparable. This could suggest that the supporter group demonstrated a strong commitment to individuals with CD.

Participants expressed their levels of concern on a scale ranging from 1 (not at all) to 4 (very much), with a mean score of 3.5. The study highlighted that the primary concern among individuals with CD was the social limitations they face, such as difficulties in eating out, participating in events or celebrations, and sharing food with others. This concern was expressed by 85.33% of the participants. The worry associated with social limitations can potentially cause individuals with CD to engage in avoidant behaviors and develop a heightened sense of being different from those without the condition. This, in turn, may lead to social withdrawal among people with CD. Additionally, these circumstances have the potential to trigger social anxiety disorder, commonly known as social phobia [21,41]. Consequently, identifying and addressing the specific concerns of individuals with CD can be instrumental in preventing the onset of anxiety disorders.

The present study found that a majority of the respondents (over 75%) rated their understanding of the disease as 3 or 4 points out of a total of 4. In a study by Halmos et al., a total of 6312 participants were surveyed about their perceived knowledge of CD and GFDs [42]. The majority of participants, persons with CD, rated themselves as having excellent knowledge (*n* = 4088; 65%) followed by good knowledge (*n* = 2006; 32%), and a small percentage rated themselves as having fair knowledge (*n* = 194; 3%). Only a few participants rated themselves as having poor (*n* = 21) or terrible knowledge (*n* = 3). The majority of participants in both studies believed that their knowledge was high, indicating a similar perception across the investigations. In the same line, Riznik et al. conducted a study in central Europe to assess the knowledge about CD among both patients and health professionals. In contrast with our self-perception assessment, the researchers found that the knowledge of both groups was deemed unsatisfactory [31]. In a study conducted by Marsilio et al., the nutritional knowledge of individuals with CD was compared to those with inflammatory bowel disease and healthy subjects [43]. The findings revealed that individuals with CD had a lower level of nutritional knowledge compared to the inflammatory bowel disease group. This could be attributed to their heightened focus on avoiding gluten in their diet, potentially causing them to overlook other crucial aspects of nutrition.

In the previously mentioned studies, it has been noted that the knowledge of individuals with CD is not ideal. However, in our study and in others, participants perceived their knowledge of the disease to be quite high. Although participants believed their knowledge to be extensive, they reported experiencing gastrointestinal and/or extraintestinal symptoms, indicating the possibility of engaging in dietary transgressions, whether voluntary or involuntary. Moreover, in the present work, participants reported actively seeking information about the condition from multiple sources, including celiac associations, healthcare professionals, and social networks. Another study also found that the most common sources of information were consistent with the present study findings [44]. Therefore, while they believed their knowledge was sufficient, there is a clear need to enhance the knowledge of this group, which would greatly benefit them.

Regarding the comfort of people with CD, in the current study participants were asked about what made them feel most uncomfortable during social gatherings. The most common response was “Ensuring that my food remains uncontaminated”, which recalls the findings of Marsilio et al., suggesting that individuals with CD prioritized avoiding cross-contact over other aspects of their diet, probably because this is an important and demanding matter of concern by itself [43].

In addition, it was noted that a significant portion of the respondents frequently or consistently informed restaurants about their illness in order to secure gluten-free choices and prevent cross-contact. Furthermore, a considerable number of individuals also took the initiative to personally inspect the cleanliness of establishments and ensure that the staff was capable of preparing a gluten-free menu. Examining these behaviors when dining out can be valuable since heightened vigilance regarding one’s diet can potentially affect the overall quality of life [16,17,45].

Considering all the above, it can be said that the presence of gluten in a diet is an important concern for these persons that was difficult enough to overlook other aspects. The present study also attempted to identify those foods or food groups people with CD were particularly cautions about. Our results indicated that these included food with extensive ingredient lists (as reported by 65% of respondents), generic foods that did not naturally contain gluten (such as dairy products, legumes, packaged vegetables, etc.) and not specifically labeled as “gluten-free” (56.33%), and spices (49.33%). They were also concerned about some non-food products, such as parapharmacy products, medicines, cosmetics, etc. (38.33%). The majority of research conducted on the gluten levels in naturally gluten-free processed foods or those labeled as certified gluten-free has indicated a relatively high occurrence of cross-contact [46,47,48]. It was observed that naturally gluten-free processed products that come in contact with gluten were supposed a greater health risk for individuals with CD compared to certified gluten-free products [46]. However, over the last years due to European regulation in terms of labeling for gluten-free foods [49], there has been a trend toward a reduction in the presence of gluten due to cross-contact in gluten-free processed foods, which may provide some reassurance to the celiac community [50]. However, it is important to note that the labeling of the presence of allergen traces is not mandatory in Europe, which is still a matter of concern for consumers with CD or allergies.

Even if gluten’s presence in the diet was the most concerning issue for the participants, other aspects that raised interest for people with CD were identified in the study. The options that obtained the highest interest were “knowing the latest news in CD research”, “CD-associated diseases”; “spices, medicines, and parapharmacy products containing gluten”, and “how to make a healthy and balanced gluten-free diet”. The significant scores in these particular categories emphasized the importance for individuals with CD to acquire more knowledge about the disease and its management. Furthermore, there was a surprisingly strong interest among this population in scientific advances and new research related to CD. This highlighted the importance of providing science-based education specifically targeted toward the population with CD. This aligns with the recommendation of ESPGHAN (European Society for Pediatric Gastroenterology, Hepatology, and Nutrition), which suggests the provision of oral and written information (leaflets, E-learnings, etc.) about the disease and the advantages of adhering to a GFD [51]. Despite previous efforts to implement such interventions [52], there is still a significant amount of work that needs to be performed in this area.

Finally, in this survey, when participants were asked if they believed that improving general knowledge about CD in the general population would enhance their quality of life, the vast majority responded affirmatively. This result indicated the importance of not only educating individuals with CD, but also targeting the general population, particularly those who have a direct impact on the daily lives of individuals with CD, such as family members, friends, and food service providers, among others.

Regarding the limitations of this study, it is important to acknowledge that the data collected were based on self-reporting, which can be influenced by respondents’ personal beliefs and opinions. Furthermore, the majority of the participants were from Europe, with limited representation from other continents. It would be valuable to include more individuals from diverse regions around the world. Additionally, the respondents in this study were active social media users, as they received the questionnaire through Instagram. It would be beneficial to investigate whether the questionnaire results are consistent across a broader population of individuals with CD, including those who do not use social media platforms. Lastly, because all questions were in closed-ended format, participants may have been influenced to select a response rather than providing their own unique answers.

On the other hand, the study has evident strengths that should be highlighted. Firstly, the sample size was considerable, as recruiting 300 participants can be challenging without a large dissemination platform. Additionally, the questionnaire used in the study was comprehensive, covering various important aspects for individuals with CD. Furthermore, this research aimed to amplify the voices of people with CD and their caregivers, as previous studies have often overlooked the perspectives of the patients themselves. Moreover, the study also sought to include the viewpoints of those who provide care for celiac sufferers, recognizing their crucial role in the health and quality of life of individuals with celiac disease.

## 5. Conclusions

It is crucial to have a comprehensive understanding of the knowledge, worries, and requirements of people with CD in order to fully grasp their situation. This understanding should encompass not only their physical wellbeing but also their emotional and social aspects. In this sense, the objective of the present work was achieved, namely to uncover the concerns and needs of people with CD. This work is the necessary starting point to define in what terms the nutritional education of these people should be carried out, as well as the gaps that should be covered by the general population to improve their quality of life. Providing targeted education on the disease and a GFD is essential for both the celiac population and the general population, as the latter plays a significant role in the daily lives of individuals with CD.

## Figures and Tables

**Table 1:** Participants’ adherence to a GFD expressed as frequency and response rates.

Adherence	Celiac (*n* = 223)		Supporters (*n* = 77)		Total (*n* = 300)	
	Frequency	Rate (%)	Frequency	Rate (%)	Frequency	Rate (%)
He/she does not follow a GFD	2	0.90	0	0.00	2	0.67
He/she follows a GFD but sometimes intentionally consumes gluten	7	3.14	4	5.19	11	3.67
He/she follows a GFD but sometimes consumes gluten unintentionally	77	34.53	16	20.78	93	31.00
He/she always follows a GFD, no exceptions	136	60.99	57	74.03	193	64.33
He/she does not know	1	0.44	0	0.00	1	0.33

No significant differences were found between the responses of the celiac group and the supporter group (*p* = 0.096 in chi-squared test).

**Table 2:** Participants’ symptoms expressed as frequency and response rates.

Types of Symptoms	Celiac (*n* = 223)		Supporters (*n* = 77)		Total (*n* = 300)	
	Frequency	Rate (%)	Frequency	Rate (%)	Frequency	Rate (%)
No symptoms	45	20.18	34	44.16	79	26.33
Extraintestinal symptoms	20	8.97	6	7.79	26	8.67
Gastrointestinal symptoms	88	39.46	28	36.36	116	38.67
Extraintestinal and Gastrointestinal symptoms	70	31.39	9	11.69	79	26.33

**Table 3:** Participants’ frequency of symptoms expressed as frequency and response rates.

Frequency of Symptoms	Celiac (*n* = 223)		Supporters (*n* = 77)		Total (*n* = 300)	
	Frequency	Rate (%)	Frequency	Rate (%)	Frequency	Rate (%)
Never	35	15.70	25	32.47	60	20.00
Very occasionally	43	19.28	24	31.17	67	22.33
A few times a year	47	21.08	12	15.58	59	19.67
Every month	41	18.38	8	10.39	49	16.33
Every week	27	12.11	2	2.60	29	9.67
Every day	30	13.45	6	7.79	36	12.00

**Table 4:** Main concerns of the participants expressed as frequency and response rates.

Concern	Celiac (*n* = 223)		Supporters (*n* = 77)		Total (*n* = 300)	
	Frequency	Rate (%)	Frequency	Rate (%)	Frequency	Rate (%)
Social limitations: eating out, attending events or celebrations, sharing food with people, etc.	191	85.65	65	84.42	256	85.33
Feeling socially excluded	58	26.01	45	58.44	103	34.33
Making the gluten-free diet healthy and balanced	90	40.36	41	53.25	131	43.67
Cross-contact	167	74.89	62	80.52	229	76.33
Following a 100% gluten-free diet	85	38.12	43	55.84	128	42.66
Other	12	5.38	6	7.79	18	6.00

**Table 5:** Situations in meetings that made people with CD feel uncomfortable expressed as frequency and response rates.

Situation	Celiac (*n* = 223)		Supporters (*n* = 77)		Total (*n* = 300)	
	Frequency	Rate (%)	Frequency	Rate (%)	Frequency	Rate (%)
Having to give explanations of CD	26	11.66	7	9.09	33	11.00
Feeling misunderstood because people underestimate the situation	52	23.32	17	22.07	69	23.00
Having to make sure that no one contaminates my food	109	48.87	43	55.84	152	50.67
Having to bring my own food	24	10.76	8	10.39	32	10.67
Other	12	5.38	2	2.60	14	4.67

**Table 6:** Sources of information of people with celiac disease expressed as frequency and response rates.

Seeking Information?	Celiac (*n* = 223)		Supporters (*n* = 77)		Total (*n* = 300)	
	Frequency	Rate (%)	Frequency	Rate (%)	Frequency	Rate (%)
Yes, through the Celiac Association., doctors, dieticians/nutritionists, and other professionals	148	66.37	50	64.94	198	66.00
Yes, through the social networks of professionals	136	60.98	48	62.34	184	61.33
Yes, through the social networks of people who talk about their experiences	79	35.43	38	49.35	117	39.00
No, I think I already know enough	8	3.59	5	6.49	13	4.33

No statistically significant differences were found between celiac and supporter participants (*p* = 0.205 in chi-squared test).

**Table 7:** Actions performed by people with celiac disease when going to a restaurant. Likert scale responses: 1 (never)–4 (always).

Action	Celiac (*n* = 223) Mean (SD)	Supporters (*n* = 77) Mean (SD)	*p* (Mann–Whitney U-Test)	Total (*n* = 300) Mean (SD)
Notify the restaurant about the disease to make sure that gluten-free food is available	3.41 (0.754)	3.17 (0.979)	0.093	3.35 (0.822)
Notify about the disease to avoid cross-contact	3.33 (0.820)	3.09 (1.028)	0.116	3.27 (0.883)
Check that the restaurant is FACE certified	2.27 (1.062)	2.06 (1.068)	0.117	2.22 (1.066)
See first-hand if the restaurant has good hygiene and can carry out a gluten-free menu	2.38 (1.112)	2.29 (1.074)	0.553	2.35 (1.101)

FACE: Federación de Asociaciones de Celíacos de España (Federation of Spanish Celiac Associations).

**Table 8:** Products that cause suspicion in people with CD expressed as frequency and response rates.

Food	Celiac (*n* = 223)		Supporters (*n* = 77)		Total (*n* = 300)	
	Frequency	Rate (%)	Frequency	Rate (%)	Frequency	Rate (%)
Gluten-free cereals	38	17.04	18	23.37	56	18.67
Generic foods that naturally do not contain gluten (dairy products, legumes, packaged vegetables, etc.) and are not labeled “gluten-free”	122	54.71	47	61.04	169	56.33
Processed food with a long list of ingredients	144	64.57	51	66.23	195	65.00
Spices	116	52.02	34	44.16	150	50.00
Non-food: parapharmacy products, medicines, cosmetics, etc.	87	39.01	28	36.36	115	38.33
Others	14	6.28	1	1.30	15	5.00

**Table 9:** Topics of interest for people with CD. Likert scale responses from 1 to 4.

Topic	Celiac (*n* = 223) Mean (SD)	Supporters (*n* = 77) Mean (SD)	*p* (Mann–Whitney U-Test)	Total (*n* = 300) Mean (SD)
I would like to learn more about what CD is, why my immune system responds to gluten intake, and what damage it causes to my body.	3.25 (0.75)	3.27 (0.72)	0.916	3.26 (0.74)
I would like to know how to make a healthy and balanced GFD.	3.36 (0.72)	3.40 (0.61)	0.923	3.37 (0.69)
I would like to know the latest news on CD research.	3.50 (0.68)	3.44 (0.72)	0.538	3.49 (0.69)
I would like to know which diseases are associated with CD.	3.42 (0.69)	3.36 (0.69)	0.484	3.40 (0.69)
I would like to hear about other experiences of people with CD.	2.86 (0.90)	2.84 (0.76)	0.769	2.85 (0.87)
I would like to learn gluten-free recipes.	3.04 (0.88)	3.16 (0.78)	0.413	3.07 (0.86)
I would like to know which spices, medicines, and parapharmacy products contain gluten.	3.43 (0.74)	3.31 (0.82)	0.233	3.40 (0.76)
I would like to know what happens if I skip the diet on a regular basis.	2.70 (1.09)	2.87 (1.09)	0.222	2.74 (1.09)
I would like to know what old age is like for a person with CD.	3.31 (0.86)	3.32 (0.77)	0.801	3.31 (0.89)
I would like to know why there could be several individuals with CD in a family.	2.85 (0.95)	2.77 (0.96)	0.541	2.83 (0.95)
I would like to know about the regulations governing the labeling of gluten-free foods.	3.29 (0.78)	3.32 (0.77)	0.725	3.30 (0.78)

**Table 10:** Participants’ perceptions about whether knowledge of the general population could improve the quality of life of people with CD.

Food	Celiac (*n* = 223)		Supporters (*n* = 77)		Total (*n* = 300)	
	Frequency	Rate (%)	Frequency	Rate (%)	Frequency	Rate (%)
No	1	0.45	0	0.00	1	0.33
Yes, considerably	208	93.27	73	94.81	281	93.67
Not sure	14	6.28	4	5.19	18	6.00

No statistically significant differences were found between celiac and supporter participants (*p* = 0.790 in chi-squared test).

## Data Availability

Data sharing is not applicable to this article.

## Appendix A

**Table A1:** Items of the questionnaire, including type of response and answer options.

*n*	Question	Type of Response	Answer Options
1	Where do you live?	Multiple choice. Only one answer can be chosen. Free response if the option “other” is chosen.	- Europe - Asia - America (north) - America (south) - Africa - Other
2	How old are you?	Multiple choice. Only one answer can be chosen.	- Under 18 years - 18 to 24 years - 25 to 44 years - 45 to 64 years - Over 65 years
3	Which gender do you most identify with?	Multiple choice. Only one answer can be chosen. Free response if the option “other” is chosen.	- Male - Female - Other
4	What is your educational level?	Multiple choice. Only one answer can be chosen.	- Primary education (preschool education, special education, and similar studies). - Secondary education (baccalaureate, secondary school, official language studies, university entrance, and similar). - Professional studies (professional training modules, first- and second-degree vocational training, intermediate and advanced vocational training cycles, other professional or artistic studies). - Medium–high education (technical engineering studies, industrial expertise, first-cycle university studies, specialization studies in secondary education, and others at the same level). - Higher education (bachelor’s degree, higher engineering, postgraduate, master’s degree, doctorate, and specialization).
5	Do you have celiac disease?	Yes/No	- Yes, I have celiac disease. - No, but I have a close person (family member, friend) who is, and that is why I am interested in the subject.
6	When were you diagnosed with the disease?	Multiple choice. Only one answer can be chosen.	- Less than a year ago - 1–5 years ago - 5–10 years ago - More than 10 years ago
7	Do you follow a strict gluten-free diet?	Multiple choice. Only one answer can be chosen.	- Yes, always, no exceptions. - Yes, although I sometimes consume it unintentionally. - Yes, although I sometimes consume it intentionally. - No, I do not follow a strict gluten-free diet. - I do not know.
8	Do you have symptoms related to celiac disease?	Multiple choice. Only one answer can be chosen.	- No, I have no symptoms. - Yes, I have gastrointestinal symptoms (diarrhea, gas, bloating, etc.). - Yes, I have extraintestinal symptoms (anemia, dermatitis herpetiformis, etc.). - Yes, I have gastrointestinal and extraintestinal symptoms.
9	If you have symptoms, how recurrent are they?	Multiple choice. Only one answer can be chosen.	- Never - Very occasionally - A few times a year - Every month - Every week - Every day
10	How would you define your level of concern about the disease?	Likert scale (1–4)	1—No concerns 4—Very concerned
11	How much do you think you know about CD?	Likert scale (1–4)	1—Nothing 4—Much
12	Are you usually looking for information about CD?	Multiple choice. More than one answer can be chosen.	- Yes, through the Celiac Association, doctors, dieticians/nutritionists, and other professionals. - Yes, through the social networks of professionals. - Yes, through the social networks of people who talk about their experience. - No, I think I already know enough.
13	What worries you most on a day-to-day basis?	Multiple choice. More than one answer can be chosen. Free response if the option “other” is chosen.	- Social limitations: eating out, attending events or celebrations, sharing food with people, etc. - Feeling socially excluded. - Making a gluten-free diet healthy and balanced. - Cross-contact. - Following a 100% gluten-free diet. - Other.
14	What makes you most uncomfortable at a social gathering?	Multiple choice. Only one answer can be chosen. Free response if the option “other” is chosen.	- Having to give explanations of CD - Feeling misunderstood because people underestimate the situation. - Having to make sure no one contaminates my food. - Having to bring my own food. - Other.
15	How often have you done these actions when going to a restaurant?-Notify the restaurant about the disease to make sure that gluten-free food is available.-Notify about the disease to avoid cross-contact.-Check that the restaurant is FACE-certified.-See first-hand if the restaurant has good hygiene and can carry out a gluten-free menu.	Likert scale (1–4)	1—Never 2—Sometimes 3—Many times 4—Always
16	What foods are you suspicious of?	Multiple choice. More than one answer can be chosen. Free response if the option “other” is chosen.	- Gluten-free cereals. - Generic foods that naturally do not contain gluten (dairy products, legumes, packaged vegetables, etc.) and are not labeled “gluten-free”. - Processed food with a long list of ingredients. - Spices. - Non-food: parapharmacy products, medicines, cosmetics, etc. - Others.
17	What do you want to know about CD?-I would like to learn more about what CD is, why my immune system responds to gluten intake, and what damage it causes to my body.-I would like to know how to make a healthy and balanced GFD.-I would like to know the latest news on CD research.-I would like to know which diseases are associated with CD.-I would like to hear about other experiences of people with CD.-I would like to learn gluten-free recipes.-I would like to know which spices, medicines, and parapharmacy products contain gluten.-I would like to know what happens if I skip the diet on a regular basis.-I would like to know what old age is like for a person with CD.-I would like to know why there could be several individuals with CD in a family.-I would like to know about the regulations governing the labeling of gluten-free foods.	Likert scale (1–4)	I am not interested at all I am a little interested I am quite interested I am very interested
18	Do you think your quality of life would improve if the general population knew more about CD?	Multiple choice. Only one answer can be chosen.	- Yes, considerably - No - Not sure
19	What is your relationship with the person with CD?	Multiple choice. Only one answer can be chosen. Free response if the option “other” is chosen.	- I live with her/him - She/he is a relative with whom I do not live - She/he is a friend - Other
20	Are you a caregiver of the person with CD (is their diet dependent on you)?	Multiple choice. Only one answer can be chosen.	- Yes - No - Sometimes
